# Energy Decomposition Scheme for Rectangular Graphene Flakes

**DOI:** 10.3390/nano14020181

**Published:** 2024-01-12

**Authors:** Hendra, Henryk A. Witek

**Affiliations:** 1 Department of Applied Chemistry, National Yang Ming Chiao Tung University, Hsinchu 30010, Taiwan; hhendra1997@gmail.com; 2 Institute of Molecular Science, National Yang Ming Chiao Tung University, Hsinchu 30010, Taiwan

**Keywords:** graphene flakes, energy decomposition, DFTB, multiple zigzag chains

## Abstract

We show—to our own surprise—that total electronic energies for a family of *m* × *n* rectangular graphene flakes can be very accurately represented by a simple function of the structural parameters *m* and *n* with errors not exceeding 1 kcal/mol. The energies of these flakes, usually referred to as multiple zigzag chains *Z*(*m*,*n*), are computed for *m*, *n* < 21 at their optimized geometries using the DFTB3 methodology. We have discovered that the structural parameters *m* and *n* (and their simple algebraic functions) provide a much better basis for the energy decomposition scheme than the various topological invariants usually used in this context. Most terms appearing in our energy decomposition scheme seem to have simple chemical interpretations. Our observation goes against the well-established knowledge stating that many-body energies are complicated functions of molecular parameters. Our observations might have far-reaching consequences for building accurate machine learning models.

## 1. Introduction

Quantum mechanical studies of large graphene flakes are obstructed to a large degree by the prohibitive cost of their calculations [1,2]. In principle, the total electronic energy of a rectangular graphene flake at its equilibrium geometry is a unique function of two parameters, *m* and *n*, corresponding, respectively, to the width and the height of the flake. Taking into account the complicated nature of this quantity and quite involved process associated with its determination—the diagonalization of the molecular Hamiltonian built from various integrals computed on an atomic basis followed by subsequent geometry optimization using gradients associated with such a non-additive many-body-like Hamiltonian—it has always been assumed that the energy function must be fairly complicated and cannot be determined in a close form as a simple function of the parameters of the model. On the other hand, in the limit m,n→∞ , a rectangular graphene flake converges toward an infinite graphene sheet, the physics of which is relatively simple, being sufficiently well described with a unit cell containing just two carbon atoms. Recent progress in the exploration of the chemical space using machine learning (ML) approaches [3,4,5,6,7,8,9,10] suggests that finding an energy function $E=E\left(m,n\right)$ can be, in fact, possible; after all, the intrinsic success of machine learning algorithms is based on the implicit existence of such a function encoded in the structure of the ML model. The explicit extraction of the quantum mechanical energy of such a system from the ML model and expressing it in a closed form as a function of the parameters *m* and *n* could be advantageous for understanding the system better and possibly for finding new important descriptors pertinent to such a formulation.

**Figure 1:**
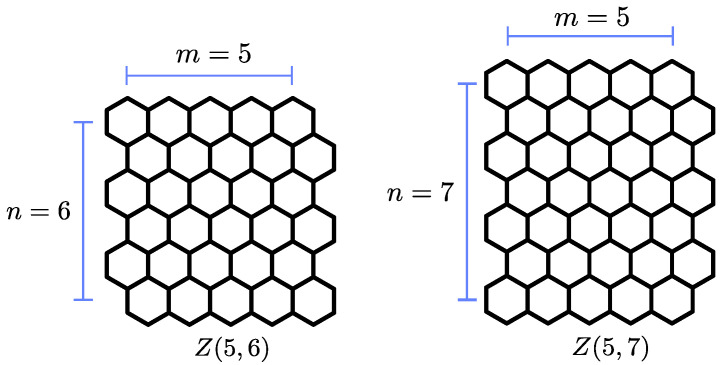
Two examples of multiple zigzag chains $Z\left(m,n\right)$ . The index *m* represents the length of the zigzag edge of these rectangular flakes, while the index *n* corresponds to the length of the armchair edge. The shape and the symmetry of the flake are slightly different for even and odd values of *n*.

In the current study, we attempt to construct such an energy function, $E\left(m,n\right)$ , restricting our attention to a single family of rectangular, hydrogen-terminated graphene flakes: multiple zigzag chains $Z\left(m,n\right)$ . (See Chapter 9 of [11,12]; for a graphical definition of these structures, see Figure 1). It is straightforward to establish that for a hydrogen-terminated multiple zigzag chain $Z\left(m,n\right)$ of width *m* and height *n*, the number of carbon atoms is equal to 2mn+2m+2n , the number of hydrogen atoms is equal to 2m+2n+2 , and the total number *N* of electrons is equal to 12mn+14m+14n . Taking into account that most quantum chemical methods require a diagonalization step [13], which scales according to $N^{3}$ with the number of electrons (see, for example, p. 191 of [1]), we can estimate that for large *m* and *n*, the cost of quantum chemical calculations for $Z\left(m,n\right)$ is proportional to $m^{3}n^{3}$ . It is therefore natural to ask whether the quantum chemical energy of a graphene flake and the corresponding equilibrium molecular geometry can be obtained by some simpler means. In the current paper, we demonstrate that indeed, for multiple zigzag chains $Z\left(m,n\right)$ , such a shortcut can be designed, and the energy function $E\left(m,n\right)$ of fully optimized multiple zigzag chains $Z\left(m,n\right)$ can be determined without quantum chemical calculations to a surprisingly high level of accuracy. We believe that a similar approach could also be designed for other carbon nanostructures, including graphene flakes [14,15] of other shapes, carbon nanotubes [16], and fullerenes [17,18].

## 2. Previous Studies

The past two decades witnessed several computational attempts to correlate wide ranges of electronic, physical, and chemical properties of molecular systems with their sizes. Schwerdtfeger and co-workers, for instance, discovered that the cohesive energies of gold [19], tin [20], and cesium clusters [21] depend on the inverse cube of the cluster size. The extrapolation of these theoretical models yielded cohesive energies with accuracy ranging from 0.05 to 0.67 eV, depending on the metal species. The total electronic and atomization energies of polyacenes (consisting of 1–8 benzene rings) were shown to change linearly with the number of benzene units and with the number of electrons [22]. The harmonic frequencies (and consequently the positions of the associated Raman lines) for acoustic-like vibrational modes in octahedral and tetrahedral nanodiamonds were shown to have inverse linear scaling with respect to the number of carbon atoms [23], allowing the determination of the size of the nanodiamonds directly from the Raman spectra. Another typical example concerns the correlation between the maximum emission wavelength of quantum dots and their sizes; it is interesting that the color of a quantum dot is determined primarily by its diameter [24,25,26], while its chemical identity plays only a lesser role. Yet another—somewhat extreme—example is constituted by the recent discovery of a simple Rydberg-like formula based on the classical theory of atoms, which is capable of predicting qualitatively the distribution and energetics of the first 10–15 atomic excited states for a wide collection of atoms [27]. These diversified examples suggest that, in many cases, simple models can provide an accuracy comparable with that of an exact quantum treatment. It is important to understand and develop such models and to analyze their strengths and limitations as in many cases their somewhat surprising and seemingly accidental numerical robustness can be explained by elementary scientific principles, some of which are yet to be discovered [28,29,30,31,32].

To the best of our knowledge, the only work on graphene flakes relevant to our investigations was performed by Gutman and his collaborators, who found that the topological resonance energies, total π -electron energies, and Dewar resonance energies of 118 catacondensed isomers of heptacene with the formula C_30_H_18_ can be represented as simple functions of either the corresponding total number of Clar covers C or the Kekulé number K [33]. In each case, a simple, few-parameter formula was found, which was capable of predicting resonance energies of all the isomers with errors no larger than 0.5%. This work was further extended to the topological resonance energies of 132 isomers of C_22_H_14_, C_24_H_14_, C_26_H_14_, C_26_H_16_, and C_28_H_16_, for which the maximal error was found to be smaller than 1.2% [34]. It is important to stress here that the fitting parameters in the energy formulas were distinct for different numbers of carbon and hydrogen atoms.

The original reason to start working on the current project was to correlate various graph-theoretical and topological descriptors of graphene flakes $Z\left(m,n\right)$ , including the Clar number Cl , the Clar count C , the Kekulé count K , and the Zhang–Zhang polynomial [35,36,37,38,39,40,41,42,43], with the total electronic energies $E\left(m,n\right)$ of these structures. Over the last decade, we have been involved in constructing a coherent mathematical theory of such invariants [44,45,46,47], so it was important for us to establish whether these topological invariants could be useful for the practical determination of the physicochemical properties of graphene flakes. In Appendix A, we show that no positive correlation exists between these quantities, suggesting that the Kekulé count K , the Clar count C , and the Clar number Cl do not constitute useful descriptors for reproducing the total electronic energies of rectangular graphene flakes $Z\left(m,n\right)$ of various sizes. However, in the process of verifying this hypothesis, we have accidentally discovered that the total energies $E\left(m,n\right)$ of the graphene flakes $Z\left(m,n\right)$ can be expressed as a surprisingly simple function of the structural parameters *m* and *n*, with most of the terms appearing in this formulation having simple chemical interpretations. In the first steps of the verification process, these simple functions of the structural parameters *m* and *n* were added to the topological fitness function in order to improve the correlation. We immediately noticed that they constitute a much better fitness function than the topological descriptors; moreover, it soon turned out that removing the topological descriptors from the fitness function does not really lower the quality of the fit. A detailed explanation of the process of constructing an optimal set of structural descriptors is discussed in the next two Sections.

## 3. Computational Methodology and Data Analysis

The molecular structures of the here-analyzed multiple zigzag chains $Z\left(m,n\right)$ with *m*, *n* = 1,…,20 were optimized using the third-order density-functional tight-binding (DFTB3) method [48] in conjunction with the 3OB parameter sets [49] using the DFTB+ 1.2 software [50]. The DFTB charge convergence criterion was selected as $10^{-12}$ a.u. Most of the studied systems displayed small or negligible HOMO-LUMO gaps. Consequently, their electronic temperature was set to either 0 K (for 153 structures corresponding to smaller values of *m* and *n*) or to 0.0001 K (for 247 quasi-metallic structures corresponding to higher values of *m* and *n*). Each optimization was performed by alternating between the steepest descent and conjugate gradient procedures in order to accelerate the calculations and to ensure convergence to a global minimum. The force convergence criterion ( $10^{-7}$ a.u.) corresponded to a very tight optimization. The resulting equilibrium geometries of the optimized flakes were planar.

It is possible that some graphene flakes may be subject to Jahn–Teller distortions, resulting in their non-planar geometry. This effect is probably strongest for flakes without hydrogen termination, where the deficiencies in chemical saturation of carbon atoms give rise to quite complicated electronic structures and, consequently, to open-shell characters and considerable degeneracies in the one-particle energy spectrum. Non-planarity deformations, allowing us to lift this degeneracy and to increase the percentage of doubly occupied levels, might be an important factor in lowering the total energy during the energy optimization process. We suspect that for the class of the hydrogen-teminated graphene flakes studied in our paper, this effect might be somewhat less pronounced owing to the well-defined, chemically saturated electronic nature of each of the flakes. An obvious way to verify whether a given flake is planar or non-planar is an inspection of the harmonic vibrational modes. Performing such a task for all the here-studied flakes is formidable due to its computational complexity. However, to answer one of the referee’s comments, we have performed DFTB+ harmonic frequency calculations for two medium-size flakes, $Z\left(10,10\right)$ and $Z\left(15,15\right)$ . All the harmonic frequencies of $Z\left(10,10\right)$ and $Z\left(15,15\right)$ turned out to be positive, showing that both these flakes are in fact planar, despite the fact that their electronic structure is metallic with 4 electrons distributed among 3 quasi-degenerate MOs with the occupation pattern 1.6:1.3:1.1 for $Z\left(10,10\right)$ and with 2 electrons distributed among 4 quasi-degenerate MOs with the occupation pattern 1.1:0.4:0.3:0.2 for $Z\left(15,15\right)$ . These two sets of calculations do not provide a proof that all the flakes studied by us are planar, but we feel that they provide quite a strong argument supporting this idea.

The set of total DFTB3 energies $\left\{E\left(m,n\right):m,n=1,\ldots ,20\right\}$ of the fully optimized structures of $Z\left(m,n\right)$ was analyzed as follows. First, we selected a family of *J* bivariate functions $A_{1}\left(m,n\right)$ , $A_{2}\left(m,n\right),\ldots ,A_{J}\left(m,n\right)$ in order to construct an energy approximation $\tilde{E}\left(m,n\right)$

(1) $$\tilde{E}\left(m,n\right)=c_{1}A_{1}\left(m,n\right)+c_{2}A_{2}\left(m,n\right)+\cdots +c_{J}A_{J}\left(m,n\right)$$

where $\left\{c_{k}\right\}$ was the set of fitting coefficients to be determined via the least-square fitting procedure. In principle, the coefficients $\left\{c_{k}\right\}$ in Equation (1) could be determined using all the available energies $E\left(m,n\right)$ in the fitting process. However, such a solution is to be avoided if one also aims at an expression applicable to larger values of *m* and *n* than those included in the fitting set. As described later in Section 4, the structures $Z\left(m,n\right)$ with low values of *m* and *n* do not closely follow the trends observed for the larger structures. Therefore, to avoid outliers in the fitting process, we decided to discard the smallest structures from our analysis. Our detailed reasoning showing how to decide which structures should be avoided in the fitting process is presented in the next section; here, we merely mention that the smallest value of *m* retained in our analysis is denoted by *M*, and the smallest value of *n*, by *N*.

The coefficients $c_{k}$ are found by minimizing the following 2-norm of the residual vector

(2) $${\left|\left|E-\tilde{E}\right|\right|}_{2}=\sqrt{\sum_{\begin{array}{c} m=M\\ n=N \end{array}}^{20}{\left(E\left(m,\;n\right)-\tilde{E}\left(m,n\right)\right)}^{2}}$$

The minimization leads to the least-square problem, which can be expressed as

(3) $$\min_{\left\{c_{k}\right\}}\left({\left|\left|E-\mathrm{Ac}\right|\right|}_{2}\right)$$

where $c={\left[c_{1},\;c_{2},\;\ldots ,\;c_{J}\right]}^{T}$ is the vector of the fitting coefficients,

(4) $$E={\left[E(M,N),\;E(M,N+1),\;\ldots ,\;E(20,20)\right]}^{T}$$

is the vector of energies used in the fitting process, and A is a (21−M)(21−N)×J matrix with the elements

(5) $$A_{(m-M)(20-N+1)+n-N+1,k}=A_{k}\left(m,n\right)$$

The least-square problem in Equation (3) was solved via the singular value decomposition (SVD) [51,52] of the matrix **A**

(6) $$A=U\Sigma V^{T}$$

which allowed us to write **c** as

(7) $$c=V\Sigma^{+}U^{T}E$$

where Σ is the diagonal matrix containing the singular values of $\sigma_{i}$ and $\Sigma^{+}$ is a diagonal matrix comprising inverses of non-vanishing singular values, $1/\sigma_{i}$ , and zeros otherwise. It was found that all the singular values $\sigma_{i}$ in Σ were always non-zero, so the inverse $\Sigma^{+}$ in Equation (7) could be computed with the full rank.

The analysis of the fitting residues is based on the vector $\Delta \epsilon =E-\tilde{E}=E-\mathrm{Ac}$ . The structure of the residual energy vector is as follows:

(8) $$\Delta \epsilon ={\left[\Delta \epsilon (M,N),\;\Delta \epsilon (M,N+1),\;\ldots ,\;\Delta \epsilon (20,20)\right]}^{T}$$

where $\;\Delta \epsilon \left(i,j\right)=E\left(i,j\right)-\tilde{E}\left(i,j\right)$ . At times, to highlight the distinct behavior of $E\left(m,n\right)$ for small values of *m* and *n*, we extend this definition to

(9) $$\Delta \epsilon ={\left[\Delta \epsilon (1,1),\;\ldots ,\;\Delta \epsilon (20,20)\right]}^{T}$$

even if the coefficients $\left\{c_{k}\right\}$ are optimized only for m∈M,…,20 and n∈N,…,20 .

## 4. Construction of the Fit

**Figure 2:**
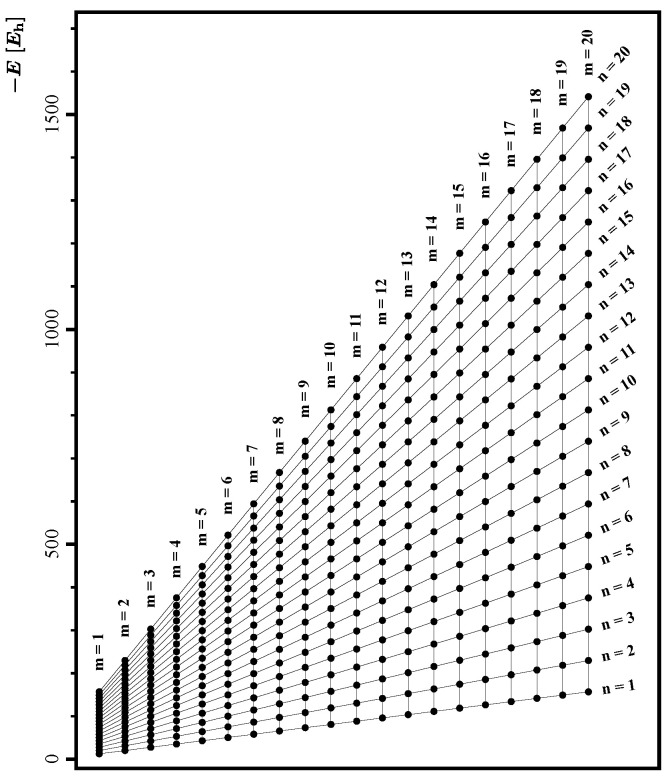
The energies $E\left(m,n\right)$ of the optimized graphene flakes $Z\left(m,n\right)$ can be arranged in the shape of a trapezoid, in which the vertical lines correspond to constant values of *m* and the diagonal lines correspond to a constant values of *n*.

As the main purpose of this research paper is to study the behavior of the energies $E\left(m,n\right)$ of the graphene flakes $Z\left(m,n\right)$ as a function of the structural parameters *m* and *n*, we start our investigation by presenting in Figure 2 a plot of $E\left(m,n\right)$ as a function of *m* and *n*. The computed energies, depicted with solid black circles, are arranged in equidistant vertical columns, each corresponding to the same value of *m*. Interestingly and surprisingly, the circles corresponding to the same value of *n* arrange themselves on straight (diagonal) lines, which for the convenience of the reader are depicted using thin black lines in Figure 2.

The highly structured form of the computed energies shown in Figure 2, resembling a trapezoidal lattice of diagonal and vertical lines, strongly suggests that the computed energies can be represented to a high level of accuracy by some analytical formula depending linearly on the parameters *m* and *n*. Therefore, in the first attempt, we chose three functions, $A_{1}\left(m,n\right)=1$ , $A_{2}\left(m,n\right)=m+n$ , and $A_{3}\left(m,n\right)=mn$ , as a basis for constructing $\tilde{E}\left(m,n\right)$ . The motivation behind this choice came from structural considerations. As mentioned previously, the number of carbon atoms is equal to $2mn+2\left(m+n\right)$ , and the number of hydrogen atoms is equal to $2\left(m+n\right)+2$ . The number of the C–H bonds is equal to $2\left(m+n\right)+2$ , and the number of the C–C bonds is equal to $3mn+2\left(m+n\right)-1$ , while the number of benzene rings in the graphene flake $Z\left(m,n\right)$ is equal to mn . All these contributions can be expressed using the three functions $A_{1}\left(m,n\right)$ , $A_{2}\left(m,n\right)$ , and $A_{3}\left(m,n\right)$ specified above. This signifies that the selected functions should be capable of capturing all the energy effects associated with atomic contributions and with contributions corresponding to the localized C–H and C–C bonds and delocalized aromatic bonds. One might alternatively treat the graphene flake $Z\left(m,n\right)$ as a macroscopic system. In this interpretation, $A_{2}\left(m,n\right)=m+n$ corresponds to the perimeter of the flake, $A_{3}\left(m,n\right)=mn$ corresponds to the area of the flake, and the choice of $A_{1}\left(m,n\right)=1$ is motivated by the finiteness aspect of each flake containing 4 corners and 4 edges, regardless of its size.

**Figure 3:**
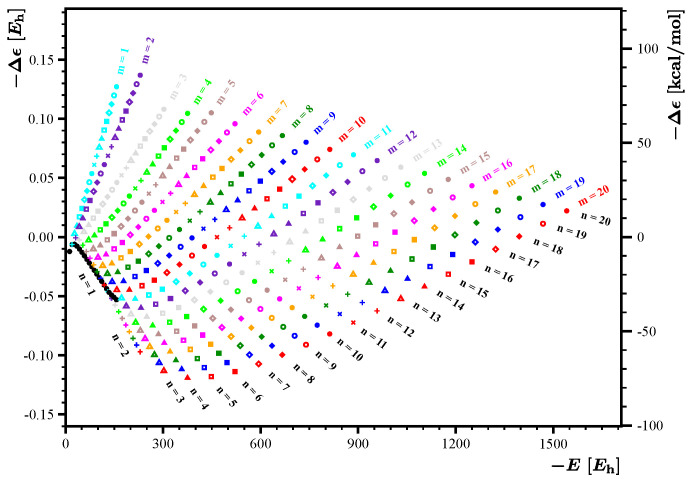
Energy residual Δϵ(m,n) corresponding to the fit obtained with a set {1,m+n,mn} . Points depicted in identical colors have identical values of *m*, and points depicted with identical symbols have identical values of *n*. For the convenience of the reader, the energy residuum scale is given in hartree (left axis) and in kcal/mol (right axis).

As anticipated, the energy expression $\tilde{E}\left(m,n\right)$ constructed using the set $\left\{1,m+n,mn\right\}$ allows us to capture most of the energy contributions, leaving out only relatively small residuals Δϵ(m,n) , whose distribution is presented graphically in Figure 3. The fit coefficients are $c_{1}=0.776231$ , $c_{2}=4.156603$ , and $c_{3}=3.436241$ ; all values are given in hartrees ( $E_{h}$ ). Even though the obtained residuals Δϵ(m,n) constitute only a tiny portion of the total energies $E\left(m,n\right)$ , for all practical purposes, the energy expression $\tilde{E}\left(m,n\right)$ constructed using the set $\left\{1,m+n,mn\right\}$ is useless because the residuals are too large and they tend to increase with increasing values of *m* and *n*. For m,n≤20 , the maximal absolute residue is about 90 kcal/mol, a value considerably larger than the 1 kcal/mol anticipated for accurate methods of quantum chemistry. On the other hand, a highly structured form of the residuals distribution shown in Figure 3 suggests that an inclusion of further fit functions in $\tilde{E}\left(m,n\right)$ can considerably improve the quality of the fit. The shape of the distribution shown in Figure 3 suggests that an appropriate fit function to be included in $\tilde{E}\left(m,n\right)$ can be expressed as $A_{4}\left(m,n\right)=m-n$ . Such a fitting function describes well the kite-shaped pattern of the distribution shown in Figure 3 and carries the information about the *m*-*n* asymmetry associated with the energy penalty corresponding to the departure of the flake from the ideal square shape.

**Figure 4:**
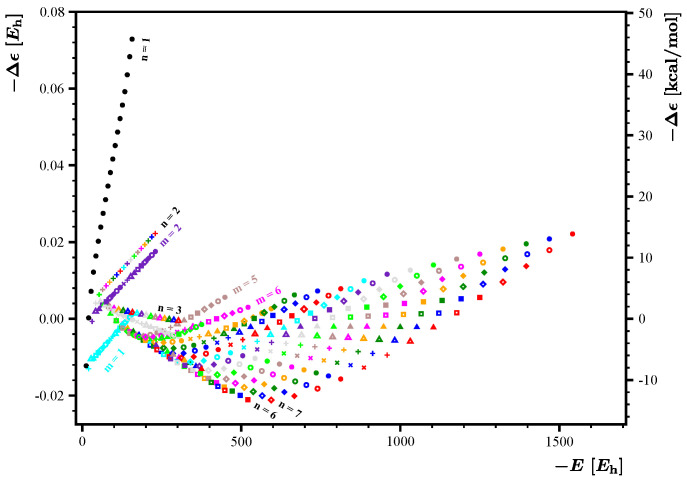
Energy residuals Δϵ(m,n) corresponding to the fit obtained with a set {1,m+n,mn,m−n} . Points depicted in identical colors have identical values of *m*, and points depicted with identical symbols have identical values of *n*. For the convenience of the reader, the energy residuum scale is given in hartree (left axis) and in kcal/mol (right axis).

The energy expression $\tilde{E}\left(m,n\right)$ constructed using the set $\left\{1,m+n,mn,m-n\right\}$ reproduces the set of energies with much better fidelity. The resulting residuals Δϵ(m,n) are presented graphically in Figure 4. The maximal positive residue is reduced from 90 kcal/mol to about 45 kcal/mol, while the maximal negative residue is reduced from −75 kcal/mol to about −15 kcal/mol. Despite the fact that the residues have been substantially reduced by the additional basis function, their magnitudes are still too large to construct a practically useful expression for $\tilde{E}\left(m,n\right)$ . Before proceeding to finding an improved expression, let us briefly discuss the important information inferred from the analysis of data in Figure 4.

 *(i)*  The fit coefficients are $c_{1}=0.776231$ , $c_{2}=4.156603$ , $c_{3}=3.436241$ , and $c_{4}=-0.006627$ $E_{h}$ . The first three coefficients are identical to the coefficients obtained with the set {1,m+n,mn} . Such a pronounced fit stability suggests that all the employed basis functions represent some physical contributions to the energy.
 *(ii)*  The energy residuals grow with increasing values of *m* and *n*. This signifies that the fitting formula cannot be extrapolated to large values of *m* and *n* without substantial loss of accuracy.
 *(iii)*  The residues corresponding to m=1–4 and n=1–3 show distinct behavior compared with the rest of residues corresponding to higher values of *m* and/or *n*.
 *(iv)*  The residues corresponding to 5≤m≤9 and to 4≤n≤6 show somewhat distinct behavior compared with the residues corresponding to higher values of *m* and *n*.

**Figure 5:**
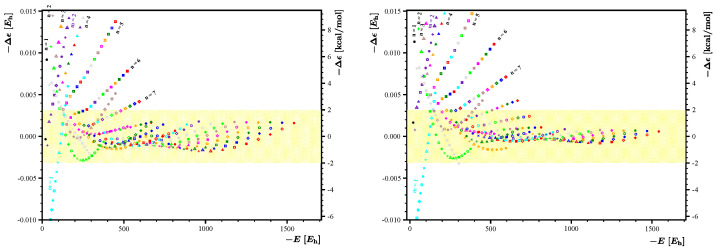
Further improvement of energy residuals obtained using the fitting set {1,m+n,mn,m−n} and M=7,N=7 (**left**) and M=10,N=10 (**right**). For the meaning of *M* and *N*, see text. The yellow background depicts region of ±2 kcal/mol corresponding to chemical accuracy.

The most important observation from the analysis given above concerns the difficulty of constructing an energy expression $\tilde{E}\left(m,n\right)$ valid at the same time for small and large values of *m* and *n*: the finite small width (height) of graphene flakes $Z\left(m,n\right)$ with m≤4 ( n≤3 ) makes these structures dissimilar to a general graphene flake $Z\left(m,n\right)$ with large values of *m* and *n*. (This effect has been partially discussed in the literature; a discovery that the ground state of linear polyacenes, i.e., the flakes $Z\left(m,1\right)$ , is an open shell singlet caused considerable stir in the chemical community at the beginning of the 2000s [53,54,55,56]). Consequently, these structures need to be studied separately and cannot be used to construct a general expression for $\tilde{E}\left(m,n\right)$ . The next several small values of *m* and *n* belong to the transitional realm, in which the finite-size effects are still partially discernible. Therefore, it is advantageous from our point of view to use in the fitting process only the energies $E\left(m,n\right)$ of the graphene flakes $Z\left(m,n\right)$ with n≥N and m≥M , where the limiting values of *M* and *N* remain yet to be determined. Motivated by this observation, we present in Figure 5 the residuals Δϵ(m,n) obtained by using the fitting set {1,m+n,mn,m−n} with N=M=7 (left panel) and N=M=10 (right panel). As expected, removing the lower portion of data with small values of *m* and *n* makes the Δϵ(m,n) magnitudes almost 10 times smaller and opens further vistas for constructing a chemically useful expression for $\tilde{E}\left(m,n\right)$ . Clearly, the results obtained with N=M=10 are more accurate with all the residuals Δϵ(m,n) with m≥6 and n≥9 falling within the window of ±2 kcal/mol of chemical accuracy.

**Figure 6:**
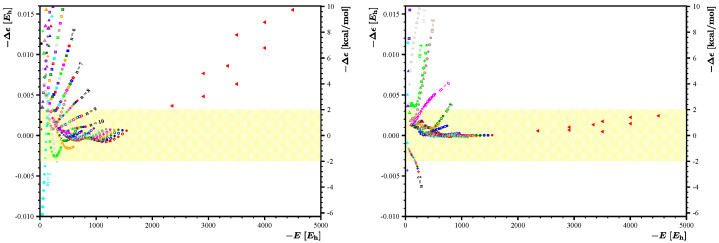
(**Left**): The residuals for the 9 new larger flakes with 25≤m,n≤35 , marked here with red triangles, show a rather large systematic departure from the chemical accuracy limit and suggest that the energy expression $\tilde{E}\left(m,n\right)$ constructed using the fitting function set B={1,m+n,mn,m−n} and 121 DFTB energy values $E\left(m,n\right)$ of smaller flakes with 10≤m,n≤20 cannot be easily extrapolated to larger structures. (**Right**): Various means to solve this problem are discussed in text. Here, we show that extending the fitting function set to $H=\{1,m+n,mn,m-n,m^{2},n^{2},n/m,m/n\}$ can substantially reduce the residuals for these 9 new larger flakes, making all of them smaller than ±2 kcal/mol. The yellow background depicts region of ±2 kcal/mol corresponding to chemical accuracy.

The usefulness of the resulting energy expression

(10) $$\tilde{E}\left(m,n\right)=0.780851+4.154860\;\left(m+n\right)+3.436457\;m\;n-0.007869\;\left(m-n\right)$$

obtained with M=N=10 was tested for its ability to predict the DFTB energies $E\left(m,n\right)$ of larger flakes $Z\left(m,n\right)$ . For this purpose, we chose 9 flakes with 25≤m,n≤35 ; for detailed definitions, see Table 1. A graphical representation of all the residuals Δϵ(m,n) computed using the energy expression $\tilde{E}\left(m,n\right)$ given by Equation (10) is displayed in Figure 6. The residuals Δϵ(m,n) of the 9 new flakes (marked with red triangles in the left panel of Figure 6) are consistently too large to fall within the chemical accuracy limit. Fortunately, the computed departure from the desired behavior is regular and suggests how to improve the optimal energy expression $\tilde{E}\left(m,n\right)$ .

In principle, there are two obvious ways to reduce the energy residuals further. The first one is based on an expansion of the fitting set by adding to it new functions of the structural parameters *m* and *n*. Since the structure of the flakes $Z\left(m,n\right)$ does not offer any further obvious insights here, we have considered—somewhat ad hoc —the following four additional functions: $m^{2}$ , $n^{2}$ , m/n , and n/m . Various expansions (labeled as *C*, *D*, *E*, *F*, *G*, and *H*) of the fitting set have been performed; their definitions and the resulting list of the fitting coefficients is given in Table 2.

As expected, the inclusion of more variables in the fitting function $\tilde{E}\left(m,n\right)$ leads to a considerable reduction in the magnitudes of the energy residuals. (See Table 1 for the detailed values.) Again, not surprisingly, the smallest magnitudes of the energy residuals for the new 9 larger flakes were obtained with the largest here-tested fitting set *H* (for a graphical representation, see the right panel of Figure 6); slightly larger values of the mean absolute error (MAE) were obtained with the fitting sets *D*, *F*, and *G*. The similarity of these magnitudes and small values of fitting coefficients for some of the fit variables (particularly for $m^{2}$ and $n^{2}$ ) allows the identification of m/n as the most important new component of the fit. The variable m/n , similarly to m−n , quantifies the departure of the flake from squareness. Its intrinsic meaning in the energy decomposition of rectangular graphene flakes and its distinction from the m−n variable remains to be understood.

**Figure 7:**
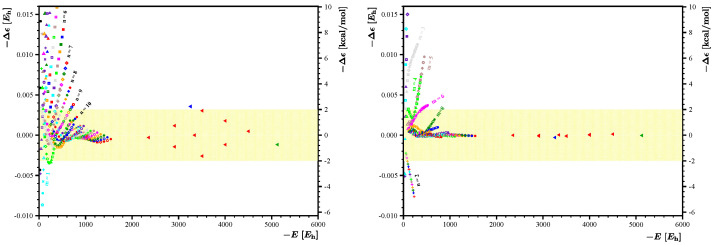
Further improvement of energy residuals obtained using the set $B^{*}=\left\{1,m+n,mn,m-n\right\}$ (left panel) and $H^{*}=\left\{1,m+n,mn,m-n,m^{2},n^{2},n/m,m/n\right\}$ (right panel). The star signifies that 121 DFTB energies $E\left(m,n\right)$ with 10≤m,n≤20 are used for the fitting process along with the 9 additional DFTB energies of the flakes listed in Table 1. The residuals for the additional points are marked with red triangles. Two new flakes, $Z\left(25,35\right)$ (with a residue marked with a blue triangle) and $Z\left(40,35\right)$ (with a residue marked with a green triangle), are used to assess the quality of the constructed energy expressions $\tilde{E}\left(m,n\right)$ . The yellow background depicts region of ±2 kcal/mol corresponding to chemical accuracy.

The second way to reduce the energy residuals further is to include the energies of some of these larger flakes in the fitting procedure. For the needs of the current analysis, we decided to include all 9 DFTB energies $E\left(m,n\right)$ of the larger flakes listed in Table 1 in the fitting set together with the previously used 121 DFTB energies $E\left(m,n\right)$ with 10≤m,n≤20 . This new fitting set was used together with the B={1,m+n,mn,m−n} and $H=\{1,m+n,mn,m-n,m^{2},n^{2},m/n,n/m\}$ fitting functions sets; the resulting fitting protocols were abbreviated as $B^{*}$ and $H^{*}$ , respectively. The list of the fitting coefficients for $B^{*}$ and $H^{*}$ is given in Table 2. The performance of these protocols is summarized in Figure 7; the left panel of this figure shows the residuals obtained with the $B^{*}$ protocol, and the right panel shows the residuals obtained with the $H^{*}$ protocol. For $B^{*}$ , including the 9 energies of the larger flakes allowed us to reduce the energy residuals to less than ±2 kcal/mol. The performance of $H^{*}$ is particularly stunning: all the energies $E\left(m,n\right)$ with 10≤m,n are reproduced with practically vanishing residuals. Since all the previously used test points were included in the fitting set, to test the quality of these two new protocols, we decided to include two new large flakes, $Z\left(25,35\right)$ and $Z\left(40,35\right)$ , in our analysis. (Note that for the 11 large rectangular flakes employed here for tests, the DFTB optimizations were performed less rigorously, with the force convergence criterion being, at most, $3\times 10^{-5}$ a.u.) Figure 7 shows that both protocols perform well for these two new flakes, but the performance of $H^{*}$ is outstanding. We feel that it is worth explicitly stating the accurate energy expression $\tilde{E}\left(m,n\right)$ constructed using the protocol $H^{*}$ ,

(11) $$\begin{array}{ccc} \tilde{E}\left(m,n\right) & = & 0.78201249+4.15429213\;\left(m+n\right)+3.43649230\;m\;n\\ & - & 0.00905570\;\left(m-n\right)+0.00000710\;m^{2}-0.00000381\;n^{2}\\ & + & 0.01061307\;m/n-0.00375723\;n/m \end{array}$$

## 5. Results and Discussion

**Figure 8:**
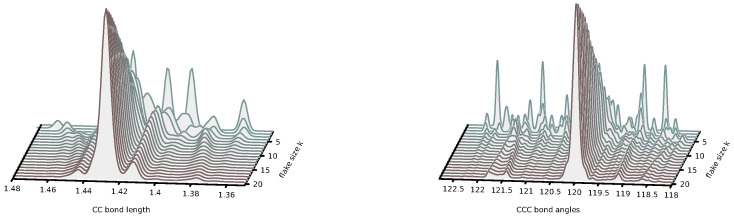
Distributions of the CC bond lengths (**left**) and CCC bond angles (**right**) in square flakes $Z\left(k,k\right)$ suggest that the graphene regime is already achieved for quite small flakes.

**Figure 9:**
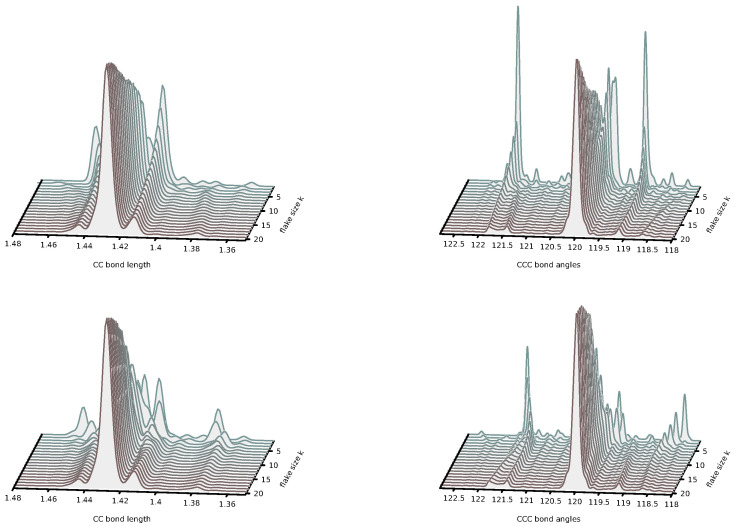
Distributions of the CC bond lengths (left panels) and CCC bond angles (right panels) in rectangular flakes $Z\left(20,k\right)$ (upper panels) and $Z\left(k,20\right)$ (lower panels) suggest that the transition to graphene-like regime is achieved faster than for square flakes.

Several interesting aspects of our work are worth highlighting:

 *(i)*  Figure 6 and the left panel of Figure 7 clearly show that the accuracy of the constructed energy expression $\tilde{E}\left(m,n\right)$ deteriorates when one extrapolates it outside of the fitting set. A similar effect is also expected for the best-constructed energy expression $\tilde{E}\left(m,n\right)$ in Equation (11) when used for very large flakes with m,n≥60 . However, as the current study shows, such a problem can be easily circumvented by adding a few new very large flakes $Z\left(m,n\right)$ with relevant values of *m* and *n* to the fitting set. For researchers interested in such extensions, we have included all the DFTB input files in the Supplementary Materials Section of this manuscript. The DFTB geometry optimization for the largest flake considered here, $Z\left(40,35\right)$ with the molecular formula C_2950_H_152_, took approximately several weeks on a 100-core computer. The reader should be aware that the optimization of a larger flake might take a considerably longer time. An interesting alternative here could be a theoretical analysis of the contributions to the total energy from the finite edge effects and possibly quantifying such an influence using non-obvious, new (m,n) -dependent basis functions. Such a development is expected to improve the description of small flakes and to permit the extrapolation of the energy formula to really large values of *m* and *n* that presently escape the possibility of direct quantum chemical calculations.
 *(ii)*  Small rectangular graphene flakes are known for their various interesting chemical deviations from the typical behavior of polycyclic hydrocarbons, including their open-shell ground state [53,54,55,56] and pronounced radical character [57,58,59,60,61,62,63]. Our work shows that larger flakes are more uniform, suggesting that the transition from finite, pyrene-like, small flakes to infinite, graphene-like, large flakes happens in the regime of m,n≈10 . To investigate this issue in more detail, we have analyzed the distribution of the CC bond lengths and CCC bond angles in the transition from small to large flakes. Before discussing the results, let us only briefly comment that in the transition from small to large flakes, one would expect that the bond lengths and bond angles would become more uniform, approaching the values corresponding to an infinite graphene sheet, i.e., CC bond lengths of 1.42–1.43 Å obtained from the DFT calculations [64,65,66,67] and of 1.42 Å obtained from experiment and an angle of $120^{\circ }$ corresponding to a hexagonal geometry. The results for square flakes defined by the formula $Z\left(k,k\right)$ for k=2 –20 are presented in Figure 8.
  These results show that the convergence to the graphene-like values is fast. In principle, the flakes $Z\left(6,6\right)$ already show distributions similar to $Z\left(20,20\right)$ . In addition to square-shaped flakes, it is interesting to check for similar convergence properties for rectangular flakes. In Figure 9, we show analogous distributions of CC bond lengths and CCC bond angles for two families of rectangular flakes, $Z\left(20,k\right)$ and $Z\left(k,20\right)$ , with values of k=2 –20. In both cases, the convergence toward the graphene-like regime is obtained faster than for the square flakes; this effect is particularly fast for the polyacene-like flakes $Z\left(20,k\right)$ . Despite the fast convergence to the graphene-like regime, the finite edge effects are clearly visible in all the distributions, suggesting that the hydrogen termination and edge effects constitute important local perturbations.
 *(iii)*  The parameters of the fit tabulated in Table 2 show surprisingly large inertias with respect to the extension of the function set with new variables. This behavior suggests that the energy decomposition has physical meaning, and its coefficients can be interpreted as sums of various energy contributions. For a flake $Z\left(m,n\right)$ , the contributions can be easily identified:
  - The energy $\epsilon_{H}$ of a hydrogen atom with multiplicity 2(m+n)+2 ;
  - The energy $\epsilon_{C}$ of a carbon atom with multiplicity 2mn+2(m+n) ;
  - The energy $\epsilon_{O}$ of an aromatic ring with multiplicity mn ;
  - The energy $\epsilon_{\mathrm{CC}}$ of a C–C bond with multiplicity 3mn+2(m+n)−1 ;
  - The energy $\epsilon_{\mathrm{CH}}$ of a C–H bond with multiplicity 2(m+n)+2 .
  Unfortunately, all these contributions involve only 3 fitting functions ( {1,m+n, and mn} ), showing that a unique determination of the five parameters $\epsilon_{H}$ , $\epsilon_{C}$ , $\epsilon_{O}$ , $\epsilon_{\mathrm{CC}}$ , and $\epsilon_{\mathrm{CH}}$ from the three coefficients $c_{1}$ , $c_{2}$ , and $c_{3}$ is not possible from our analysis. In the future studies, we plan to extend our analysis to other structured graphene flakes, including prolate rectangles Pr(k,m,n) [11,68], oblate rectangles Ob(m,n) [11,69], and hexagons O(k,m,n) [11,46]. We expect that the distinct dependence of the total energy on the structural parameters for these structures will help to uniquely determine the five parameters $\epsilon_{H}$ , $\epsilon_{C}$ , $\epsilon_{O}$ , $\epsilon_{\mathrm{CC}}$ , and $\epsilon_{\mathrm{CH}}$ defined above.
 *(iv)*  The energies used to construct the energy expression given by Equation (11) in addition to the usual size and shape dependence encoded by the parameters *m* and *n* also include contributions related to the geometry relaxation effects. In our study, all these components are treated collectively. It would be very interesting to consider the relaxation effects individually, for example, by starting the geometry optimization from a rectangular patch of an idealized infinite graphene sheet with uniform hydrogen terminations. The relaxation effects can be divided into three types of contributions: (1) those corresponding to the relaxation of the carbon sublattice, (2) those corresponding to the relaxation of the hydrogen sublattice, and (3) those corresponding to the synergic relaxation of both lattices simultaneously. Such an analysis is beyond the scope of the current study.
 *(v)*  Numerous studies tried to correlate various topological invariants with the energetic stability of polycyclic aromatic hydrocarbons. The vast efforts of the graph-theoretically oriented chemists over the last few decades have created substantial literature on this topic. (Probably the best existing account describing the body of results on the Kekulé count K is the monography of Cyvin and Gutman [11]; the results on other invariants are scattered throughout the literature.) Our study may show that such an effort might be somewhat superfluous as similar effects can be achieved more readily by correlating the energetics with appropriate structural parameters (and their simple algebraic functions) of the whole family of analyzed structures.

## 6. Conclusions

We have computed the DFTB energies $E\left(m,n\right)$ of rectangular graphene flakes $Z\left(m,n\right)$ with 1≤m,n≤20 at their optimized geometries. The resulting set of energies has been used to construct an approximate energy expression $\tilde{E}\left(m,n\right)$ for $E\left(m,n\right)$ in a form of a linear combination of various simple functions of the structural parameters *m* and *n*. In order to construct an accurate energy expression $\tilde{E}\left(m,n\right)$ , several important factors have been explicitly considered in our work:

 *(i)*  It has been recognized that small graphene flakes $Z\left(m,n\right)$ with 1≤m,n≤9 are structurally dissimilar to larger flakes due to their finite-size effects. Those structures have been excluded from the fitting set, which finally comprised 121 energies of flakes $Z\left(m,n\right)$ with 10≤m,n≤20 .
 *(ii)*  It has been noted that in order to be able to extrapolate the energy expression $\tilde{E}\left(m,n\right)$ outside of the fitting region, we need to include in the fitting set several larger structures $Z\left(m,n\right)$ with 20≤m,n .
 *(iii)*  The set of fitting functions resulting from structural considerations and comprising physically meaningful variables {1,m+n,mn,m−n} needs to be expanded to $\left\{1,m+n,mn,m-n,m^{2},n^{2},n/m,m/n\right\}$ . The physical interpretation of these new variables remains to be understood.
 *(iv)*  Performance tests of the energy expression $\tilde{E}\left(m,n\right)$ have been performed using two additional graphene flakes, $Z\left(25,35\right)$ and $Z\left(40,35\right)$ .

The best approximation to $E\left(m,n\right)$ was produced using a fitting protocol abbreviated as $H^{*}$ (for details, see Table 2); the explicit form of the resulting $\tilde{E}\left(m,n\right)$ is given by Equation (11). This expression is very accurate and falls withing the requirement of chemical accuracy (i.e., the energy residuals Δϵ(m,n) are smaller than ±2 kcal/mol) for all graphene flakes $Z\left(m,n\right)$ with 10≤m,n≤20 used in the current study; in fact, all the relevant residuals are almost negligible (for details, see the right panel of Figure 7).

## Figures and Tables

**Table 1:** Energy residuals $\left|\Delta \epsilon (m,\;n)\right|$ for larger graphene flakes calculated using the energy expression $\tilde{E}\left(m,n\right)$ and constructed for various fitting function sets with M=N=10 . Definitions of the fitting sets ( B–H ) are given in Table 2.

*m*	*n*	$\left|\Delta \epsilon (m,\;n)\right|$ (kcal/mol)						
		B	C	D	E	F	G	H
25	25	2.3	13.0	0.35	0.39	0.32	0.43	0.36
30	30	3.0	14.8	0.23	1.54	0.92	1.04	0.42
35	35	4.8	15.7	1.51	0.59	0.30	0.24	0.65
26	30	5.4	17.2	0.92	0.85	0.83	0.86	0.84
30	26	4.0	17.2	0.05	3.26	1.92	1.64	0.30
27	35	7.8	18.9	3.66	2.55	0.35	1.80	1.09
35	27	6.8	10.0	0.44	2.77	1.75	1.94	0.92
31	35	8.8	10.8	2.68	0.65	0.84	0.08	1.41
35	31	9.7	12.9	1.72	1.49	1.57	1.45	1.53
MAE		5.8	17.8	1.28	1.57	0.98	1.05	0.84

**Table 2:** The values of the coefficients $c_{k}$ (in $E_{h}$ ) calculated using various fit protocols. The last row gives a decimal multiplier for each type of coefficient.

Set	M,N	$A_{k}\left(m,n\right)$							
		1	m+n	mn	m−n	−m2	−n2	m/n	−n/m
A	1	7.7623	4.1566	3.4362	−…	−…	−…	*…*	−…
B	1	7.7623	4.1566	3.4362	−6.6270	−…	−…	*…*	−…
B	7	7.8123	4.1550	3.4364	−7.7102	−…	−…	*…*	−…
B	10	7.8085	4.1549	3.4365	−7.8689	−…	−…	*…*	−…
$B^{*}$	10	7.8835	4.1543	3.4365	−7.8967	−…	−…	*…*	−…
C	10	7.7803	4.1551	3.4365	−7.6720	−1.3128	−…	*…*	−…
D	10	7.9091	4.1542	3.4365	−7.1670	−…	−4.6793	*…*	−…
E	10	7.8809	4.1544	3.4365	−6.9701	−1.3128	−4.6793	*…*	−…
F	10	7.7718	4.1544	3.4365	−8.4538	−1.3128	−0.3942	1.0152	−…
G	10	7.9420	4.1543	3.4364	−7.8008	−1.5278	−4.6793	*…*	−5.6842
H	10	7.8329	4.1544	3.4365	−9.2846	−1.5278	−0.3942	1.0152	−5.6842
$H^{*}$	10	7.8201	4.1543	3.4365	−9.0557	−0.7103	−0.3812	1.0613	−3.7572
		( $\times 10^{-1}$ )	( $\times 10^{\;0}$ )	( $\times 10^{\;0}$ )	− ( $\times 10^{-3}$ )	− ( $\times 10^{-5}$ )	− ( $\times 10^{-5}$ )	( $\times 10^{-2}$ )	− ( $\times 10^{-3}$ )

**Figure A1:**
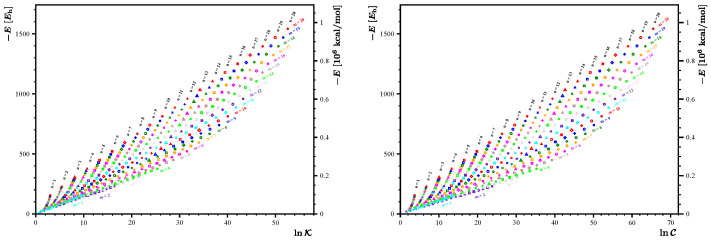
The energies $E\left(m,n\right)$ of the optimized graphene flakes $Z\left(m,n\right)$ with 1≤m,n≤20 plotted against the natural logarithm of the corresponding Kekulé count $K\equiv K\left(m,n\right)$ (**left**) and Clar count $C\equiv C\left(m,n\right)$ (**right**). The shapes of the resulting distributions show more complexity than the similar dependence on *m* and *n* plotted in Figure 2. For a fixed value of *m*, the energies $E\left(m,n\right)$ change linearly with the values of $K\left(m,n\right)$ and $C\left(m,n\right)$ . For a fixed value of *n*, the energies $E\left(m,n\right)$ exhibit an approximate cubic dependence on the values of $K\left(m,n\right)$ and $C\left(m,n\right)$ .

**Figure A2:**
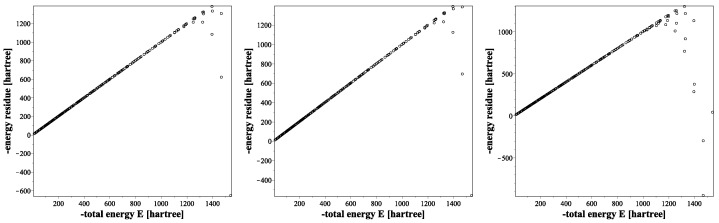
Fitting the DFTB energies $E\left(m,n\right)$ directly to the Kekulé count K and the Clar count C produces very large energy residuals. The fitting sets are $\left\{1,K\right\}$ for the (**left**) panel, $\left\{1,C\right\}$ for the (**middle**) panel, and $\left\{1,K,C\right\}$ for the (**right**) panel.

**Figure A3:**
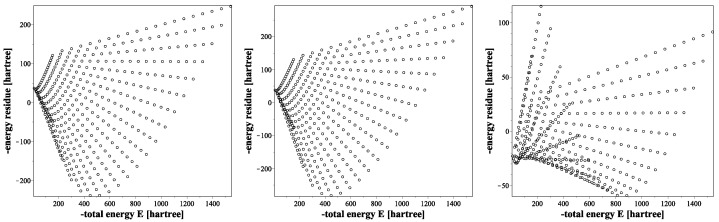
Fitting the DFTB energies $E\left(m,n\right)$ to the natural logarithm of the Kekulé count K and the Clar count C produces a much better fit than the direct fit to K and C shown in Figure A2. Nevertheless, the resulting energy residuals are still huge (approximately 1000 times larger) in comparison to a fit constructed using the structural parameters *m* and *n* and their simple algebraic functions. The fitting sets are $\left\{1,\ln K\right\}$ for the (**left**) panel, $\left\{1,\ln C\right\}$ for the (**middle**) panel, and $\left\{1,\ln K,\ln C\right\}$ for the (**right**) panel.

## Data Availability

The detailed list of 411 rectangular graphene flakes used in this study is given in file DFTB_energies.txt together with their optimized DFTB energies. Molecular structures of the 411 DFTB optimized flakes are given in the zipped directory DFTB_geometries. DFTB input files corresponding to this set of output geometries are given in the zipped directory DFTB_input. These files and directories can be retrieved from the zipped archive located in the Supplementary Material section of this paper.

## Supplementary Materials

The following supporting information can be downloaded at: https://www.mdpi.com/article/10.3390/nano1010000/s1, The list of 411 rectangular graphene flakes together with their optimized DFTB energies is given in file DFTB_energies.txt. Molecular structures of these flakes are given in the zipped directory DFTB_geometries. DFTB input files are given in the zipped directory DFTB_input.

## Abbreviations

The following abbreviations are used in this manuscript:

ML	Machine learning
DFTB3	Third-order density-functional tight-binding
HOMO	Highest occupied molecular orbital
LUMO	Lowest unoccupied molecular orbital
SVD	Singular value decomposition

## Appendix A. Correlations between Topological Invariants and Total Electronic Energies

The Kekulé count K and Clar count C of a multiple zigzag chain graphene flake $Z\left(m,n\right)$ can be computed directly from the Zhang–Zhang polynomial $\mathrm{ZZ}\left(Z\left(m,n\right),x\right)$ of $Z\left(m,n\right)$ . (See Equation (21) of [70] and Equations (7) and (8) of [71].) We have

(A1)
$$\begin{array}{ccc} K\equiv & K\left(m,n\right)= & \mathrm{ZZ}\left(Z\left(m,n\right),0\right) \end{array}$$

(A2)
$$\begin{array}{ccc} C\equiv & C\left(m,n\right)= & \mathrm{ZZ}\left(Z\left(m,n\right),1\right) \end{array}$$

For an even number of zigzag chains, the ZZ polynomial of $Z\left(m,2n\right)$ is given by the following determinantal expression [46]

(A3)
ZZZm,2n,x=⋮w0w1w2⋯wn−1w−1w0⋱⋱⋮⋮0⋱⋱⋱w2⋮⋱⋱⋱w1⋮0⋯0w−1w0

where

(A4)
$$w_{l}\equiv w_{l}\left(x\right)=\sum_{j=0}^{2}\left(\begin{array}{c} 2\\ j \end{array}\right)\left(\begin{array}{c} m+l\\ 2l+j \end{array}\right){\left(1+x\right)}^{l+j}$$

An analogous expression for the ZZ polynomial of $Z\left(m,2n+1\right)$ can be found in [46]. (See Equation (39) of [46] for the determinantal expression and Equations (28) and (29) of [46] for the matrix elements. Note that the meaning of the indices *m* and *n* in the current work and in [46] is reversed.) Note also that the determinantal expressions for the Kekulé count K of $Z\left(m,2n\right)$ and $Z\left(m,2n+1\right)$ first appeared as Equations (14.34) and (14.35), respectively, in the monography of Cyvin and Gutman [11].

In contrast to the quite involved determinantal expressions for K and C , the value of the Clar number Cl of $Z\left(m,n\right)$ is very simple:

(A5)
$$Cl\equiv Cl\left(m,n\right)=n$$

This fact follows directly from the interface theory of benzenoids developed by Langner and Witek [72]. (A detailed discussion on how to determine the Clar number using the interface theory of benzenoids is given in Section 5.3 of [73].) In particular, the multiple zigzag chains $Z\left(m,n\right)$ belong to the family of regular *n*-tier benzenoids, and for every regular *n*-tier benzenoid, the Clar number Cl is equal to the number of vertices in the Hasse diagram associated with a given *n*-tier benzenoid. For multiple zigzag chains $Z\left(m,n\right)$ , this particular poset is a fence (or a zigzag poset) with *n* elements (see the right bottom corner of Figure 8 of [74]), so consequently, Cl=n .

### Appendix A.1. Energies E(m,n) as Functions of lnK and lnC

A distribution of the DFTB energies $E\left(m,n\right)$ and their correlation with the Kekulé count K and Clar count C is presented in Figure A1. Both distributions are more complicated than the almost doubly linear relationship with *m* and *n* shown previously in Figure 2. For fixed *m*, $E\left(m,n\right)$ depends approximately linearly on $\ln K\left(m,n\right)$ , while for fixed *n*, this dependence is approximately cubic. Similar relationships also hold for $C\left(m,n\right)$ .

### Appendix A.2. Energy Residuals for Various Fitting Sets Involving Only Topological Invariants

Figure A1 suggests that the DFTB energies $E\left(m,n\right)$ depend logarithmically on the topological invariants $K\left(m,n\right)$ and $C\left(m,n\right)$ . It is to be expected that no valuable relations can be obtained by a direct fit of $E\left(m,n\right)$ to $K\left(m,n\right)$ and $C\left(m,n\right)$ . Indeed, Figure A2 confirms these expectations, showing that $K\left(m,n\right)$ and $C\left(m,n\right)$ cannot be used directly to construct a useful energy approximation $\tilde{E}\left(m,n\right)$ .

As expected, the correlation of the DFTB energies $E\left(m,n\right)$ with lnK and lnC is much better. The energy residuals from fitting $E\left(m,n\right)$ to the sets $\left\{1,\ln K\right\}$ , $\left\{1,\ln C\right\}$ , and $\left\{1,\ln K,\ln C\right\}$ are shown in Figure A3. The residuals show a large degree of structure, but their numerical values are approximately 1000 times larger than the residuals obtained by a fit to a set {1,m+n,mn} of simple structural parameters. Motivated by this observation, we discontinued working with the topological parameters K and C in this study, focusing entirely on fitting sets comprising simple algebraic functions of the structural parameters *m* and *n*. Note that the Clar number Cl being equal to *n* is effectively accounted for in the development presented in the main body of this paper.
